# L-carnitine alleviated acute lung injuries induced by potassium dichromate in rats: involvement of Nrf2/HO-1 signaling pathway

**DOI:** 10.1016/j.heliyon.2021.e07207

**Published:** 2021-06-09

**Authors:** Abeer Salama, Hany M. Fayed, Rania Elgohary

**Affiliations:** a Pharmacology Department, National Research Centre, El- Buhouth St., Dokki, Cairo, 12622, Egypt; b Narcotics, Ergogenics and Poisons Department, National Research Centre, El- Buhouth St., Dokki, Cairo, 12622, Egypt

**Keywords:** L-carnitine, Chromium, Acute lung injury, Nrf2, Keap1, HO-1, NQO1, GCLM

## Abstract

The activation of the Nrf2/HO-1 signaling pathway regulates cellular antioxidant stress and exerts anti-inflammatory and cytoprotective effects against acute lung injury (ALI). The present study aimed to evaluate the therapeutic role of L-carnitine (LC) against potassium dichromate (PD) - induced acute lung injury in adult male albino rats via modulation of Nrf2/HO-1 signaling pathway. For this purpose, forty rats were randomly allocated into 5 groups (8 rats each). The normal group received intranasal (i.n.) saline, while the ALI group received intranasal instillation of PD as a single dose of 2 mg/kg. The 3d – 5th groups received PD then after 24 h administered L-carnitine (25, 50 and 100 mg/kg; orally) for 3 consecutive days. The therapeutic effect of L-carnitine was evaluated by assessment of serum levels of glutathione (GSH) and malondialdehyde (MDA) along with measurement of lung contents of transforming growth factor β1 (TGFβ1), protein kinase B (AKT), Nuclear factor erythroid-2 related factor 2 (Nrf2), Kelch-like ECH-associated protein 1 (Keap1), heme oxygenase-1 (HO-1), NAD(P)H quinone oxidoreductase 1 enzyme (NQO1) and glutathione cysteine ligase modifier subunit (GCLM) expression. Post-treatment with L-carnitine effectively increased the levels of GSH and AKT, elevated Nrf2 and its target genes and decreased the levels of MDA and TGFβ1 in comparison with PD control rats. Additionally, L-carnitine effectively reduced the number of goblet cell, inhibited the mucus formation in bronchioles and interstitial inflammatory infiltrate as well as alleviated the destruction of alveolar walls, and the congestion of blood vessels in lung tissue induced by PD. Our findings showed that L-carnitine may be a promising therapeutic agent against PD-induced acute lung injury.

## 1 Introduction

Acute lung injury (ALI) or its extreme manifestation, acute respiratory distress syndrome (ARDS), is an acute inflammatory lung injury which associated with high morbidity and mortality along with the progression of multiple organ failure [1]. More than 3 million patients suffer from ARDS annually, and they represent up to 10% of ICU patients [2]. The main pathological features of ALI include diffuse alveolar epithelial damage, pulmonary vascular endothelial cells, neutrophils infiltration and influx of protein-rich fluid into the alveolar spaces [3, 4]. The potential mechanisms of ALI may include inflammatory reactions, apoptosis, redox imbalance [5] and goblet cell hyperplasia (GCH) [6].

The wide use of chromium (Cr) in industries leads to release of huge amount of toxic Cr compounds in the environment. The trivalent form of chromium (Cr III) is less toxic and insoluble while hexavalent form (Cr VI) is usually linked with oxygen and more toxic [7, 8]. Cr VI can generate reactive oxygen species (ROS) during their reduction, and these ROS can cause injury to the cellular proteins, lipids, and DNA [9]. The role of oxidants and oxidative injury in the pathogenesis of ALI/ARDS in experimental studies and in studies of patients with ALI/ARDS is well documented [10]. An imbalance between generation and removal of ROS via antioxidant defense system can lead to oxidative stress [11].

Nuclear factor E2-related factor 2 (Nrf2) plays a vital role in elimination of ROS and in inhibition of lung diseases induced via oxidative stress [12]. Under quiescent conditions, Nrf2 is bound to Kelch-like ECH-associated protein 1 (Keap1) in the cytoplasm. Meanwhile, under stressed conditions Nrf2 is dissociated from Keap1, translocated into the nucleus, and bound to antioxidant response elements (AREs) in the promoter region of cytoprotective antioxidant enzymes, such as heme oxygenase-1 (HO-1) [13]. The activation of the Nrf2/HO-1 signaling pathway also inhibits the activation of Nod-like receptor protein 3 (NLRP3), thus reducing IL-1β expression and exerting anti-inflammatory and cytoprotective effects [14].

L-carnitine (β-hydroxy-γ-trimethyl-amino-butyric acid) is biosynthesized largely in the kidney, liver, and brain [15] and transported by organic cation transporters (OCTNS) that localized in the lung, trachea, brain, heart, intestine, kidney, and liver [16]. L-carnitine has a role in cell membrane stabilization, metabolism of branched chain amino acids, and scavenging of the free radicals that is reported in many *in vitro* and *in vivo* studies, thus protecting antioxidant defense system from oxidative stress [17]. Additionally, L-carnitine has antioxidant and anti-inflammatory activities [18]. For this purpose, we aimed to explore the possible therapeutic role of L-carnitine against PD induced acute lung injury via modulation of Nrf2/HO-1 signaling pathway.

## 2 Materials and methods

### 2.1 Animals

Wister albino male rats of 140–150 g were provided by the Animal House of the National Research Centre (Cairo, Egypt). The rats were group-housed under temperature- and light-controlled conditions (24 ± 2 °C under a 12 h light/dark cycle) and had free access to standard laboratory rodent chow and water. The animal experiments were performed in accordance with the guidelines of the Institutional Animal Ethics Committee (Medical Research Ethics Committee (MREC) of the NRC, Cairo, Egypt.

### 2.2 Chemicals and kits

L-carnitine was purchased from MEPACO, Egypt. PD was purchased from Sigma Aldrich Chemical Co. (USA). Reduced glutathione (GSH), catalase, malondialdehyde (MDA) were obtained from Biodiagnostic kits, Egypt. TGF-β, Nrf2 and Keap1 ELISA kits were obtained from specific ELISA kit, SinoGeneClon Biotech Co., Ltd, China.

### 2.3 Experimental design

Forty rats were randomly allocated into five groups (n = 8). The 1st group received i.n. saline and served as the normal group, while the 2nd group received PD intranasally as a single dose of 2 mg/kg and served as a model of acute lung injury group [19]. 3^rd^-5^th^ groups received PD intranasally then after 24h received L-carnitine (25, 50 and 100 mg/kg; orally) for 3 days.

### 2.4 Determination of oxidative stress biomarkers

At the end of experiment, blood samples were withdrawn from rats of all groups via retro-orbital vein under light ether anesthesia [20]. Blood was allowed to coagulate and then centrifuged at 3000 rpm for 15 min [21]. Serum was used for estimation of reduced glutathione (GSH) and malondialdehyde (MDA) levels. The GSH estimation method is based on the reduction of 5,5 dithiobis (2-nitrobenzoic acid) (DTNB) with reduced GSH to produce a yellow compound. The reduced chromogen is directly proportional to GSH concentration and its absorbance can be measured at 405 nm by using a commercial kit was used (Biodiagnostic, Egypt) [22]. MDA estimation method depends on the formation of MDA as an end product of lipid peroxidation which reacts with thiobarbituric acid producing thiobarbituric acid reactive substance (TBARS), a pink chromogen, which can be measured spectrophotometrically at 532 nm, an MDA standard was used to construct a standard curve against which readings of the samples were plotted [23].

### 2.5 Determination of Nrf2, Keap1 and TGF-β

The animals were sacrificed by decapitation under light anesthesia [24]. Lung was washed with saline and placed in ice-cold phosphate buffer (pH 7.4) to prepare 20% homogenate using a tissue homogenizer (MPW-120, BitLab Medical instruments, Poland). Homogenized tissues were centrifuged at 4000 rpm/min for 10 min at 4^ο^C using a cooling centrifuge (Laboratory Centrifuge, 2 K15, Sigma Co., Germany). The supernatant was collected and stored at -80 °C and then used for estimation of lung contents of Nrf2, Keap1 and TGF-β expression.

Lung contents of Nrf2, Keap1 and TGF-β were determined using ELISA kit (NOVA kit, Beijing, China). Standards and samples were pipetted into wells with immobilized antibodies specific for rat Nrf2, Keap1 and TGF-β and then were incubated for 30 min at 37C. After incubation and washing, horseradish peroxidase-conjugated streptavidin was pipetted into the wells and incubated for 30 min at 37C, which were washed once again. Tetramethylbenzidine (TMB) substrate solution was added to the wells and incubated for 15 min at 37C; a color was developed proportionally to the amount of Nrf2, Keap1 and TGF-β bound. Color development was discontinued (stop solution) and after 10 min color intensity was measured at 450 nm [25].

### 2.6 Real-time PCR quantification of NQO1, GCLM, HO-1 and protein kinase B (Akt)

Total RNA was extracted from 30 mg of tissue samples according to manufacture instructions (RNA Extraction kit (#K0731, Termo Scientifc, Lithuania)). Extracted RNA concentration was quantifed using Nanodrop spectrophotometry (Quawell 5000, USA); then 110 ng of total RNA transcribed using RNA reverse transcriptase kits ((#K0251) (Termo Scientifc, Lithuania)).The thermal cycler was programmed at 25 °C for 10 min, 37 °C for 120 min, 85 °C for 5 min, and 4 °C for 20 h. Prepared cDNA, was used in the qPCR analyzer (Step One, Applied Biosystems, Singapore) using the MAXIMA SYBR Green qPCR Master Mix with the following program: 1 cycle at 95 °C for 10 min; 40 cycles of 95 °C for 15 s, 60 °C for 30 s and 72 °C for 30 s; one cycle at 95 °C for 15 s, 60 °C for 1 min and 95 °C for 15 s. The specific primers of NQO1 (Forward primer 5′CAGCGGCTCCATGTACT-3, Reverse primer ′ 5′ GACCTGGAAGCCACAGAAG-3′) & housekeeping GAPDH.

Total RNA was extracted from the Lung samples using TRIzol reagent (Takara Bio, Inc.) and treated with RNase-free DNase (Sangon Biotech, Co., Ltd., Shanghai, China) to remove genomic DNA contamination. Next, 1 μg RNA was reverse transcribed to cDNA using a reverse transcription system kit (Sangon Biotech Co., Ltd.). Gene expression level of GCLM was evaluated by qPCR. Primers for GCLM were as follows: Forward, 5′-CTGTACCAGTGGGCACAGGTAA-3′ and reverse, 5′-TTGGGTCATTGTGAG TCAGTAGC-3'. The mRNA expression level of β-actin was also detected as an internal control for each sample. qPCR was performed using a SYBR Green I PCR kit (Takara Bio, Inc.), according to the manufacturer's instructions, in an ABI PRISM 7500 sequence detection system (PerkinElmer, Norwalk, CT, USA). Amplification conditions were as follows: 95 °C for 10 s, followed by 40 cycles of 95 °C for 5 s and 60 °C for 41 s.

The mRNA expression level of HO-1 and Akt gene was assessed using real-time PCR standardized by co-amplification with the housekeeping B-Actin for HO-1 and GAPDH gene for Akt as an internal control. HO-1 and Akt RNA was extracted from lung tissue using Trizol reagent. RNA was reverse-transcribed using M-MLV reverse transcriptase (Invitrogen, Carlsbad, CA, USA) and then used for PCR with specific primers. Quantification of HO-1 was carried out by using HO-1 RT-PCR fluorescence diagnostic kit Cat. No. M.R. 246187 according to manufactures' instructions. For amplification, 40 cycle of 95ᵒ C for 5s, and 65ᵒ C for 30s, 72ᵒ C for 30s, then 1 min at 60ᵒ and 2 min at 72ᵒ C were performed with HO-1 forward primer 5′ ATGGCCACCCTGATCCACATC-3′,HO-1 reverse primer 5′ TGTTGCGCTCAATCTCCTCCT-3′, using Rotor-Gene Q5 plex real-time Rotary analyzer (Corbettlife sciences, USA).Quantification of Akt was carried out by using Akt RT-PCR fluorescence diagnostic kit Cat. No. A.B 517302 according to manufactures' instructions. For amplification, 40 cycle of 95ᵒ C for 5s, and 61ᵒ C for 1s, 72ᵒ C for 30s, then 1 min at 60ᵒ and 10 min at 72ᵒ C were performed with Akt forward primer 5′-GTGGCAAGATGTGTATGAG-3′, Akt reverse primer5′-CTGGCTGAGTAGGAGAAC-3′, using Rotor-Gene Q5 plex real-time Rotary analyzer (Corbettlife sciences, USA).

### 2.7 Histopathological examination

The different lung lobes tissues from each animal were fixed in10% neutral buffered formalin, then washed, dehydrated, and embedded in paraffin blocks. Sections of 5μm thick were stained with hematoxylin and eosin (H&E), for histopathological examination. Five Lung sections per group were examined. Ten random high microscopic fields (X40) per section were scanned for assessment of the histopathological lesions using binocular Olympus CX31 microscope. The paraffin-embedded lung specimens were sectioned and stained with PAS stain (Sigma-Aldrich Chemical) to assess goblet cell hyperplasia as a measure of airway mucus hypersecretion [26]. The stained sections were examined, and five images were taken for each section. The goblet cell density counts were reported as cells per high power field (GC/HPF). A quantitative morphometric scoring of mucus hyper-secretion, interstitial inflammatory infiltrate, and congested, thrombosed blood vessels with peri-vascular inflammatory infiltrate was performed. Each item was scored 0–3 (0 = normal; 1 = mild; 2 = moderate; 3 = severe) [27].

### 2.8 Statistical analysis

All experiments were performed in triplicates and the results were presented as mean ± SE. The statistical significance of differences was calculated and analyzed by one way analysis of variance (ANOVA), followed by followed by Tukey's multiple comparisons test using GraphPad Prism 5 Software version 5 (SanDiego, CA). Values of p < 0.05 were considered to be significant.

## 3 Results

### 3.1 Effect of L-carnitine on oxidative stress markers

**Figure 1:**
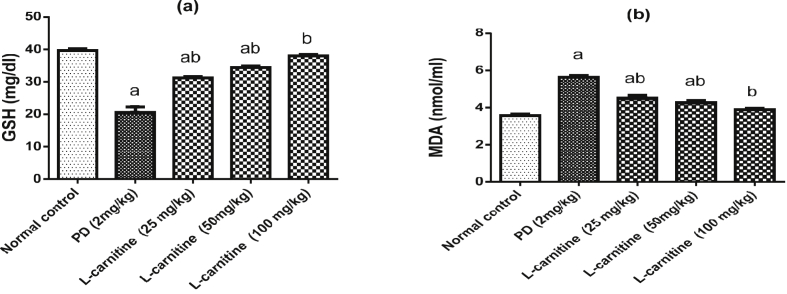
Effect of treatment with L-carnitine on serum (a) GSH and (b) MDA levels in rats subjected to PD - induced acute lung injury. Values are expressed as the mean ± SE and were analyzed using one-way ANOVA with Tukey’s multiple comparisons test. ^a^Significant at P < 0.05 when compared with control group. ^b^Significant at P < 0.05 when compared with PD group.

Rats were treated with PD showed significant decrease in serum GSH level with marked increase in MDA level as compared with normal control group. Meanwhile, treatment with L-carnitine (25, 50 and 100 mg/kg) showed a significant increases in serum GSH levels by 52%, 67% and 85% respectively, with marked decrease in MDA levels by 19.5%, 24 % and 31% respectively, in comparison to PD control group. Treatment with L-carnitine 100 mg/kg returned serum GSH and MDA levels to their normal values (Figure 1a, b).

### 3.2 Effect of L-carnitine on lung Nrf2 and Keap1 expression

**Figure 2:**
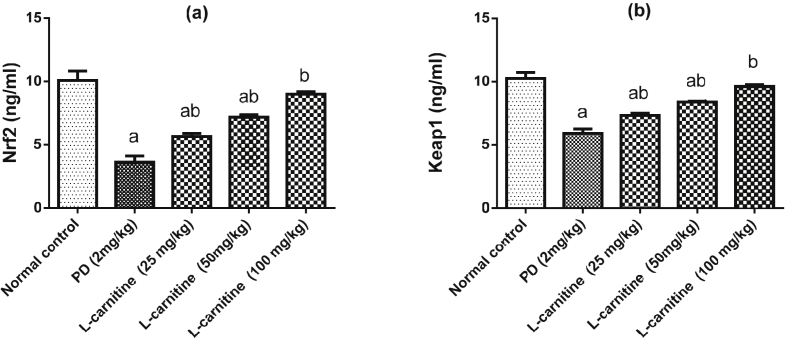
Effect of treatment with L-carnitine on lung (a) Nrf2 and (b) Keap1 in rats subjected to PD - induced acute lung injury. Values are expressed as the mean ± SE and were analyzed using one-way ANOVA with Tukey’s multiple comparisons test. ^a^Significant at P < 0.05 when compared with control group. ^b^Significant at P < 0.05 when compared with PD group.

Lung Nrf2 and Keap1 significantly decreased in rats treated with PD as compared with normal control group. Treatment with L-carnitine (25, 50 and 100 mg/kg) significantly corrected these parameters by 56 %, 98 % and 148% and 24 %, 42 % and 63% respectively, as compared to PD treated rats. Treatment with L-carnitine 100 mg/kg returned tissue Keap1 to its normal values (Figure 2a, b).

### 3.3 Effect of L-carnitine on TGF-β1 expression

**Figure 3:**
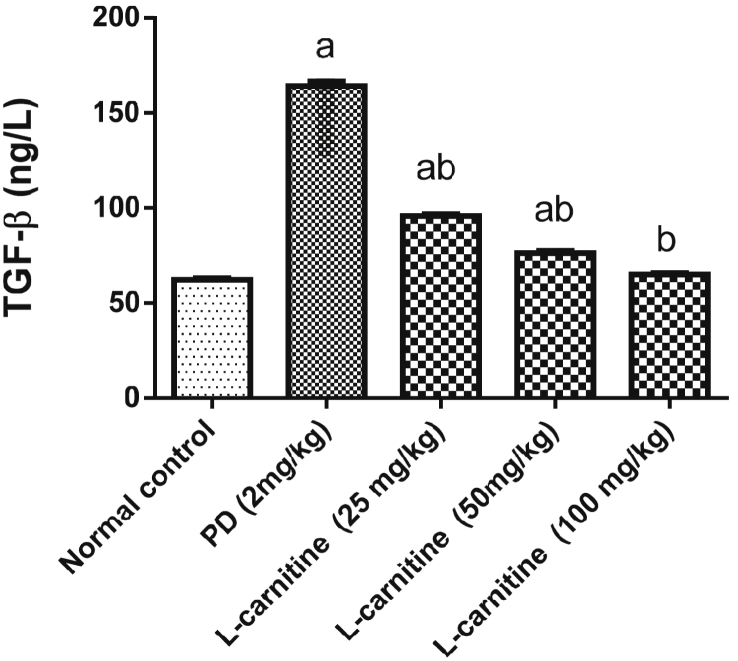
Effect of treatment with L-carnitine on lung TGF-β1 in rats subjected to PD - induced acute lung injury. Values are expressed as the mean ± SE and were analyzed using one-way ANOVA with Tukey’s multiple comparisons test. ^a^Significant at P < 0.05 when compared with control group. ^b^Significant at P < 0.05 when compared with PD group.

The expression of lung TGF-β1 was increased in PD control group as compared to normal control group. Administration of L-carnitine (25, 50 and 100 mg/kg) resulted in marked decreases in tissue activities of TGF-β1 by 42%, 54% and 60 % respectively, as compared to PD -treated group. Treatment with L-carnitine 100 mg/kg returned tissue activities of TGF-β1 to their normal values (Figure 3).

### 3.4 Effect of L-carnitine on lung HO-1 and AKT expression

**Figure 4:**
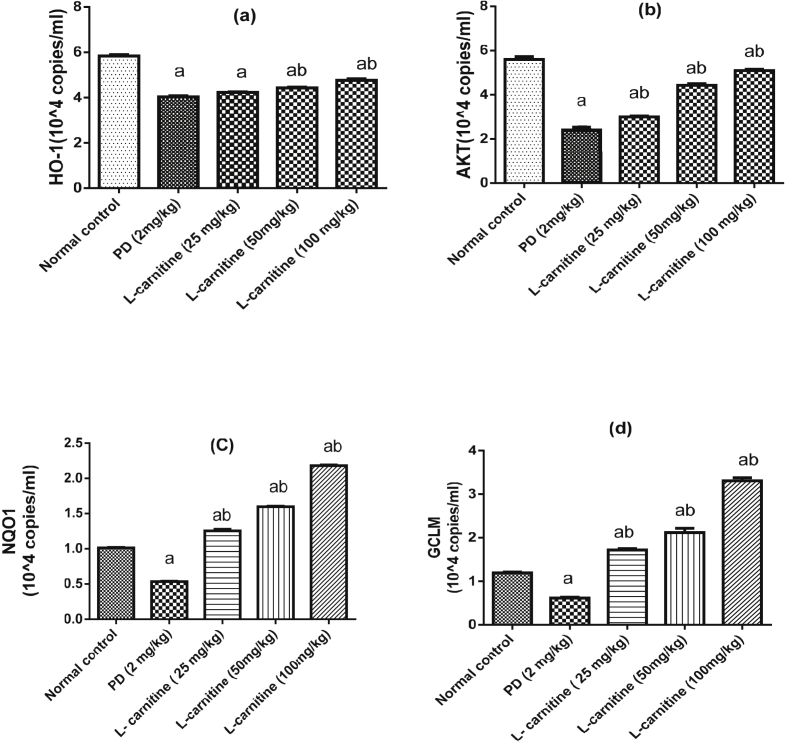
Effect of treatment with L-carnitine on lung (a) HO-1 and (b) AKT (c) NQO1 (d) GCLM mRNA expressions in rats subjected to PD - induced acute lung injury. Values are expressed as the mean ± SE and were analyzed using one-way ANOVA with Tukey’s multiple comparisons test. ^a^Significant at P < 0.05 when compared with control group. ^b^Significant at P < 0.05 when compared with PD group.

It was observed that PD administration considerably inhibited the expression levels of HO-1 and AKT in lung tissues as compared to normal group. However, L-carnitine (50 and 100 mg/kg) significantly enhanced the expression of HO-1 by 10 % and 17% respectively, as compared to PD treated rats. Moreover, treatment with L-carnitine (25, 50 and 100 mg/kg) significantly enhanced the expression of AKT by 25 %, 84% and 113 % as compared to PD treated rats (Figure 4a, b).

### 3.5 Effects of L-carnitine on target genes of Nrf2 (NQO1 and GCLM)

PD significantly decreased expression of NQO1 and GCLM by 47.2% and 48.3% as compared to normal control group. All doses of L-carnitine produced a significant increase in NQO1 mRNA expression level by 133%, 198 % and 307 % and GCLM mRNA expression level by 179%, 243% and 446 % when compared to PD group (Figure 4c, d).

### 3.6 Histopathological study

**Figure 5:**
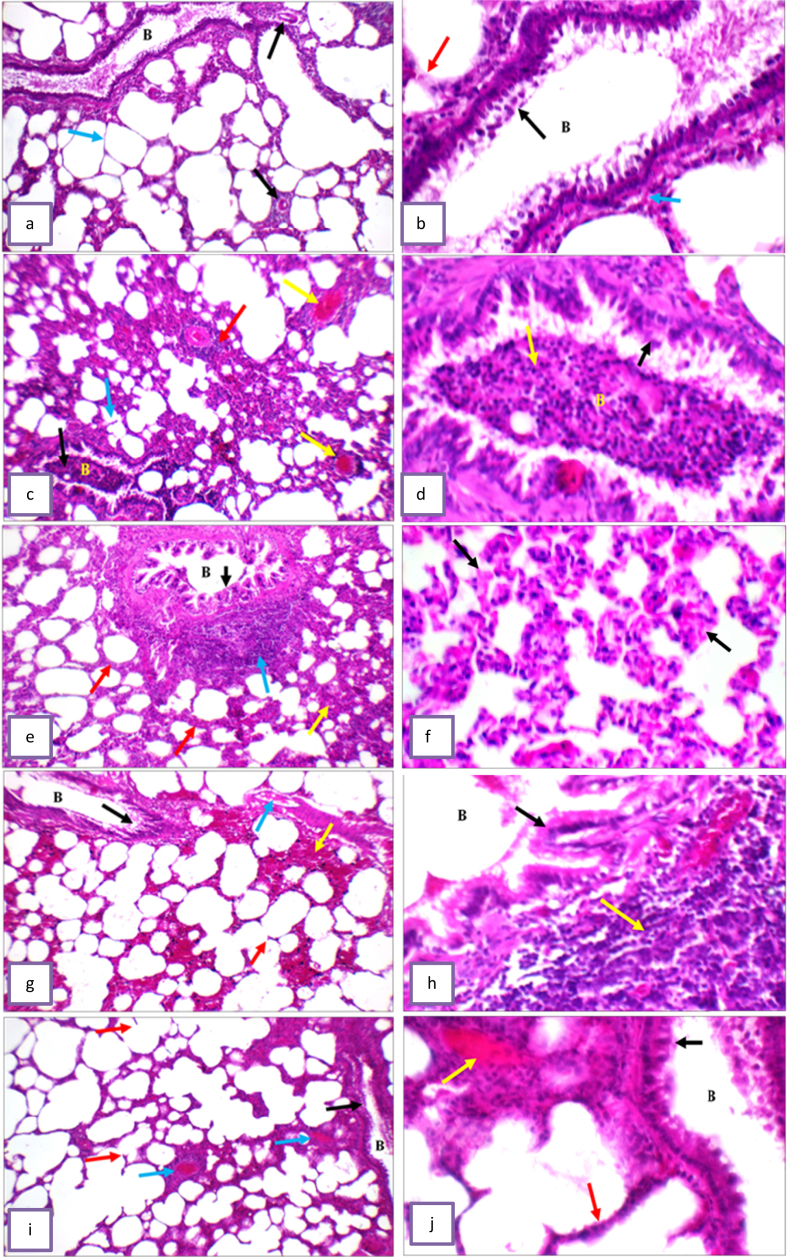
Effect of treatment with L-carnitine on lung histopathology. (a) Lung sections from normal control rats showed average bronchioles (B), average alveolar walls (blue arrow), and average interstitium with average interstitial blood vessels (black arrow). (b) High power view from normal control rats showed bronchiole (B) with average epithelial lining (black arrow), average alveolar walls (red arrow), and average interstitium. (C) lung sections from PD treated group showed average bronchioles (B) filled by mucous entangling inflammatory cells (black arrow), destructed alveolar walls (blue arrow), marked interstitial inflammatory infiltrate (red arrow), and thrombosed blood vessels (yellow arrow). (d) High power view from PD treated group showed markedly thickened alveolar walls (black arrow), and thick-walled congested blood vessels (blue arrow) with marked peri-vascular inflammatory infiltrate (yellow arrow). (e) Lung sections from PD and L-carnitine 25 showed average bronchioles (B) with average epithelial lining (black arrow) and marked peri-bronchiolar inflammatory infiltrate (blue arrow), mildly thickened alveolar walls (red arrow), and mildly expanded interstitium (yellow arrow). (f) High power view from PD and L-carnitine 25 showed mildly thickened and edematous alveolar walls (black arrow). (g) Lung sections from PD and L-carnitine 50 showed average bronchioles (B) with average epithelial lining (black arrow), mildly destructed alveolar walls (red arrow) with interstitial hemorrhage (yellow arrow), and mildly congested blood vessels (blue arrow). (h) High power from PD and L-carnitine 50 showed average bronchioles (B) with average epithelial lining (black arrow) and marked peri-bronchiolar inflammatory infiltrate (yellow arrow). (i) Lung sections from PD and L-carnitine 100 sowed average bronchioles (B) with average epithelial lining (black arrow), mildly destructed alveolar walls (red arrow), and mildly expanded interstitium with few thrombosed blood vessels (blue arrow). (j) High power from PD and L-carnitine 100 showed average bronchioles (B) with average epithelial lining (black arrow), average alveolar walls (red arrow), and mildly expanded interstitium with few thrombosed blood vessels (yellow arrow). H&E pictures from left column is 200 X, right column is 400 X magnification.

**Figure 6:**
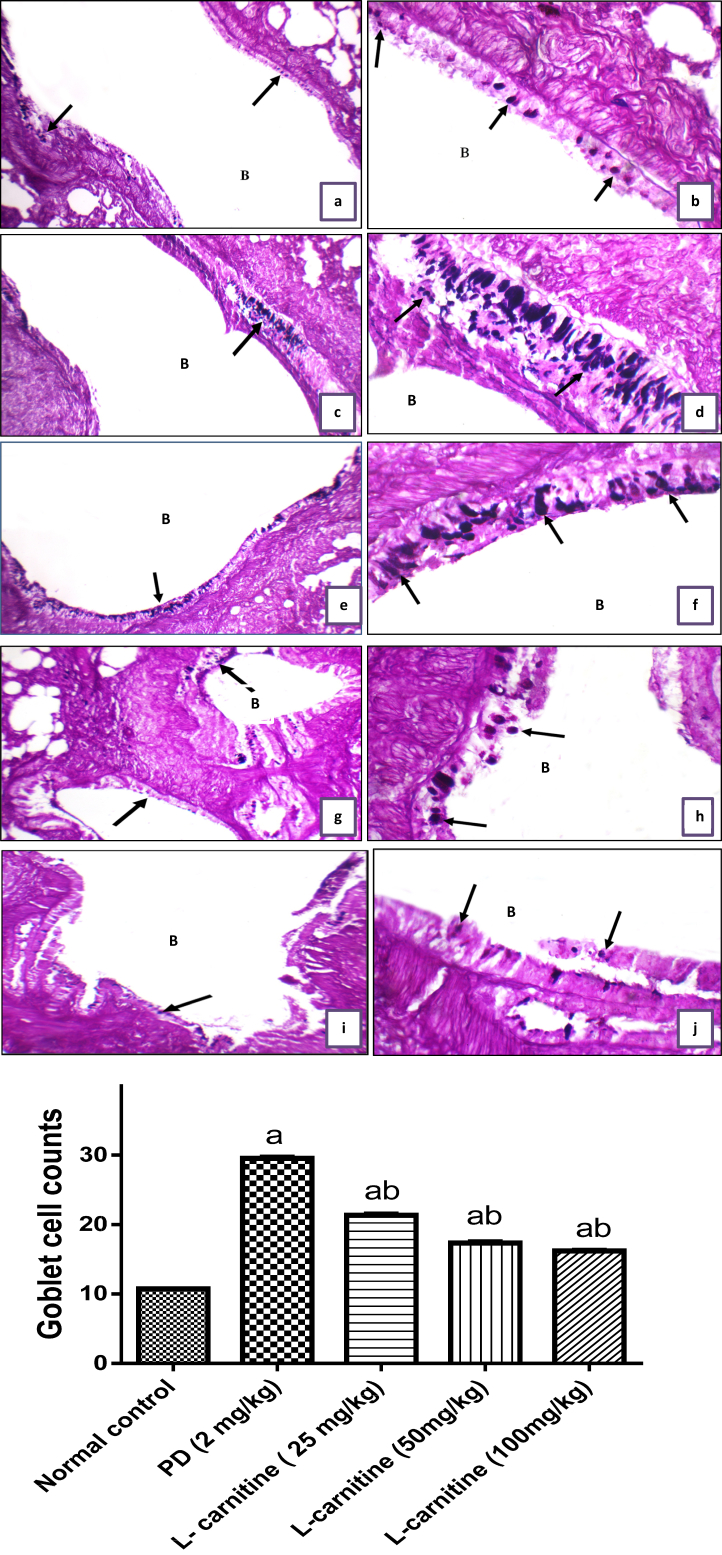
Effect of L-carnitine on goblet cell counts. (a) lung showing bronchioles (B) with average distribution of goblet cells (black arrow) normal control. (b) High power view showing bronchioles (B) with average distribution of goblet cells/HPF (black arrow) normal control. (C) Lung showing bronchioles (B) with excess distribution of goblet cells (black arrow) PD treated group. (d) High power view showing bronchioles (B) with excess distribution of goblet cells/HPF (black arrow) PD treated group. (e) Lung showing bronchioles (B) with moderate distribution of goblet cells (black arrow) PD and L-carnitine 25. (f) High power view showing bronchioles (B) with moderate distribution of goblet cells /HPF (black arrow) PD and L-carnitine 25. (g) Lung showing bronchioles (B) with mild distribution of goblet cells (black arrow) PD and L-carnitine 50. (h) High power view showing bronchioles (B) with mild distribution of goblet cells/HPF (black arrow) PD and L-carnitine 50. (i) Lung showing bronchioles (B) with mild distribution of goblet cells (black arrow) PD and L-carnitine 100. (j) High power view showing bronchioles (B) with mild distribution of goblet cells/HPF (black arrow) PD and L-carnitine 100. PAS-Alcian blue stain picture, left column is 200 X, right column is 400 X magnification. Morphometric analysis of goblet cell counts. ^a^Significant at P < 0.05 when compared with control group, ^b^Significant at P < 0.05 when compared with PD group.

**Figure 7:**
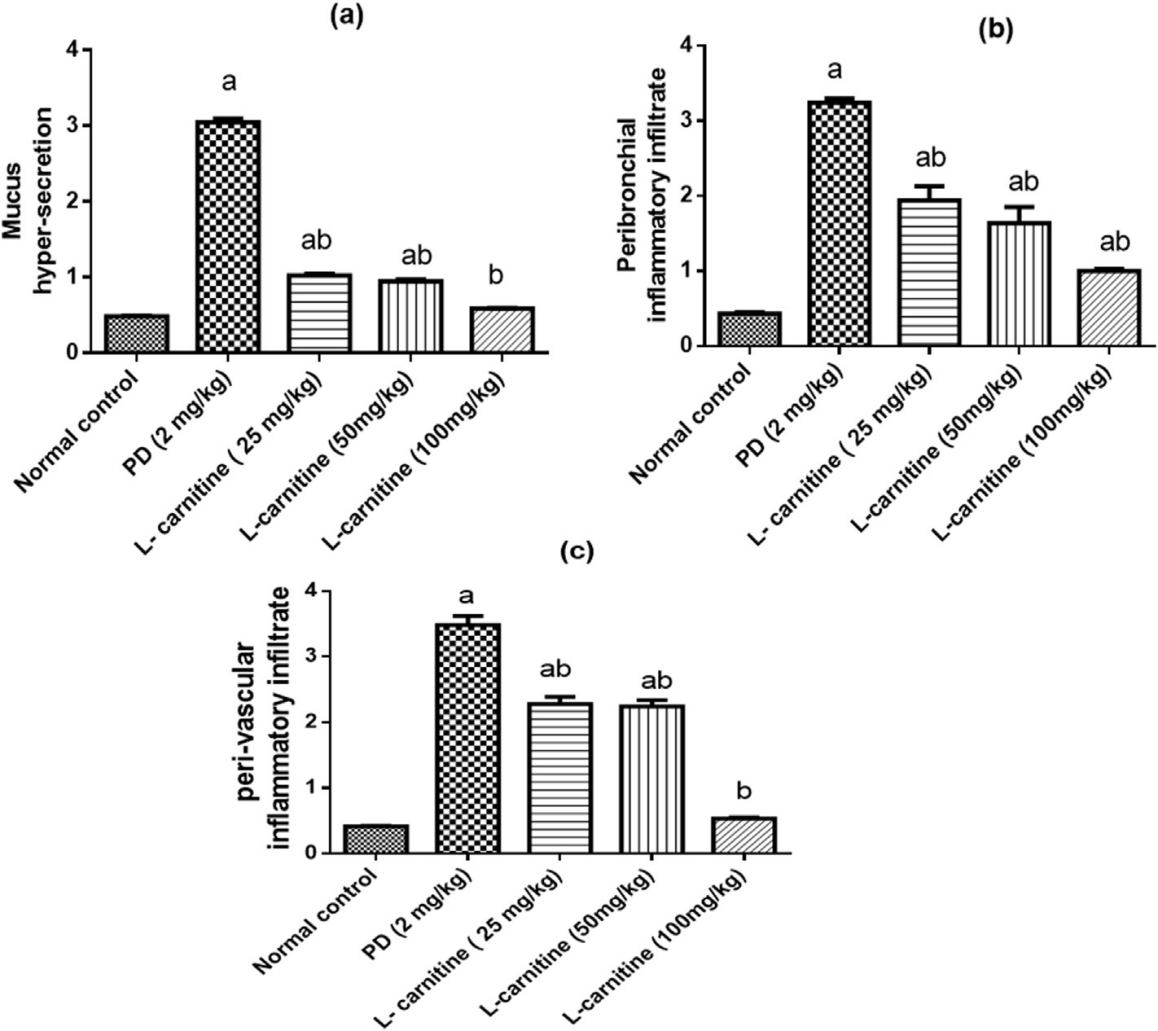
Effect of treatment with L-carnitine on lung histopathological alteration scoring. (a) Mucus hyper-secretion (b) Peribronchial inflammatory infiltrate (c) peri-vascular inflammatory infiltrate. Values are expressed as the mean ± SE and were analyzed using one-way ANOVA with Tukey’s multiple comparisons test. ^a^Significant at P < 0.05 when compared with control group. ^b^Significant at P < 0.05 when compared with PD group.

Histological picture of lung sections from normal control rats showed normal lung structure. Normal bronchioles and blood vessels were found between lung alveoli which have thin interalveolar septa. High power showed bronchiole (B) with average epithelial lining, average alveolar walls, and average interstitium (Figure 5 a, b). The lung of (PD treated group) showed bronchioles with average epithelial lining, filled by mucous entangling inflammatory cells, markedly destructed alveolar walls, marked interstitial inflammatory infiltrate, and markedly congested and thrombosed blood vessels with peri-vascular inflammatory infiltrate. High power showed markedly thickened alveolar walls, and thick-walled congested blood vessels with marked peri-vascular inflammatory infiltrate (Figure 5 c, d). In (PD and L-carnitine treated at dose of 25 mg/kg) lung sections showed average bronchioles with average epithelial lining and marked peri-bronchiolar inflammatory infiltrate, mildly thickened and edematous alveolar walls, mild interstitial inflammatory infiltrate, and mildly congested with few thrombosed blood vessels. High power showed mildly thickened and edematous alveolar walls (Figure 5 e, f). In (PD and L-carnitine treated at dose of 50 mg/kg) lung showed average bronchioles with average epithelial lining and marked peri-bronchiolar inflammatory infiltrate, mildly destructed and markedly edematous alveolar walls, and thick-walled congested blood vessels with marked peri-vascular inflammatory infiltrate. High power showed average bronchioles (B) with average epithelial lining and marked peri-bronchiolar inflammatory infiltrate (Figure 5 g, h). In (PD and L-carnitine treated at dose of 100 mg/kg) lung showed average bronchioles with average epithelial lining and mild peri-bronchiolar inflammatory infiltrate, mildly destructed and mildly thickened alveolar walls with mildly congested alveolar blood vessels, mildly expanded interstitium with few thrombosed blood vessels. High power showed average bronchioles (B) with average epithelial lining, average alveolar walls, and mildly expanded interstitium with few thrombosed blood vessels (Figure 5 i, j). Distribution of goblet cells in different groups and goblet cell counts presented in Figure 6. The severity of alteration in the lung was blindly scored microscopically, and scores are presented in Figure 7.

## 4 Discussion

The current work explored therapeutic role of L-carnitine against acute lung injury induced by chromium in rats. Chromium is extensively exist in industries and wastes that pollute soil and drinking water [28]. Hexavalent chromium is a very soluble powerful oxidizing agent to the biological systems causing mutagenic, teratogenic, and carcinogenic effects [29]. It can generate ROS during their reduction, which in turn cause injury to the cellular DNA, proteins and membrane lipids [9]. Oxidative stress induces generation of superoxide radicals and hydrogen peroxide, which promotes oxidative damage to cellular constituents lipid and protein [30]. Antioxidants play the vital role in scavenging of ROS, so they prevent the biological membranes from damage [15].

Results of the present study clearly show that intranasal instillation of PD caused significant depletion of GSH and marked increase of MDA which is mainly attributed to oxidative stress caused by Cr VI. Our results are in consistent with the previous studies which showed that Cr VI induced oxidative stress by increasing MDA as well as decreasing GSH, SOD and CAT anti-oxidant enzyme activities in rats [31, 32]. Meanwhile, treatment with L-carnitine significantly protected the cellular constituents via declining MDA and elevating GSH levels, which was previously proven [15]. Additionally, increased nicotinamide adenine dinucleotide phosphate hydrogen (NADPH) production might be the mechanism where L-carnitine could restore GSH levels [33]. As well, L-carnitine could suppress free radical formation and protected tissues from injury by mending lipid peroxidation which confirms our findings [34].

To further explore the mechanism of the therapeutic effects of L-carnitine against ALI, the expression of several markers were assessed. Nrf2 has a fundamental role in protecting the cells against oxidative damage induced by metal toxicants [35]. The anti-oxidant role of Nrf2 is primarily attributed to the expression of genes that antagonize oxidative stress. Previous research has explored that pharmacological activation of Nrf2 could alleviate ALI severity [36]. Upon stresses conditions via oxidative stress, Nrf2 activates and dissociates from Keap1, then translocates into the nucleus to activate the antioxidant target genes [37] such as HO-1 and NQO1 detoxifying enzymes [38]. In the present study, exposure to PD significantly inhibited Nrf2/Keap1/NQO1 expression in the lung tissues. Our findings are in accordance with previous study that showed involvement of Nrf2 pathway in chromium-induced lung injury [39]. PD disrupted the association of Nrf2/Keap1 in the nucleus and provokes ROS production that inversely correlated with Nrf2 expression and its target genes Ho-1 and detoxification gene NQO1 [40]. Treated groups with L-carnitine showed upregulation of Nrf2/Keap1/NQO1 expression which proposed that Nrf2/Keap1/NQO1 activation is implicated in the therapeutic effect of L-carnitine against acute lung injury. Previous studies have shown that L-carnitine upregulated Nrf2/Keap1 expression *in vitro* and in *vivo* [41], which confirmed our findings. Nrf2 act as a transcription factor that initiates the expression of several key anti-oxidant genes, one of these genes include HO-1 [37]. In addition, Nrf2 transcription factor contributes to COPD susceptibility [42], and induces the expression of the glutathione cysteine ligase catalytic subunit (GCLC) and the glutathione cysteine ligase modifier subunit (GCLM) that are involved in GSH synthesis [43]. The mechanisms underlying L-carnitine-associated upregulation of GSH and GSH-dependent detoxifying enzymes via GCL are unknown. The current results revealed, for the first time, that the reduction in GCLM expression associated with a reduction in GSH levels after PD injection, while L-carnitine induced Nrf2 transcription elevated GCLM expression with GSH *s*ynthesis.

Nrf2 dissociation from Keap1 and translocation to the nucleus is initiated via Nrf2 phosphorylation [44]. Intracellular phosphatidylinositol kinase (PI3K) is linked with oxidative damage and apoptosis [45]. Activated PI3K phosphorylates AKT that stimulates Nrf2 activation to translocate into the nucleus and consequently induce anti-oxidant genes expression [35]. HO-1 activation seems to be an endogenous defensive mechanism used by cells to diminish inflammation and tissue damage in several injury models. Upon translocation of Nrf2 to the nucleus, it binds to Maf then Nrf2/Maf bind to ARE to promote transcription of HO-1 [46]. It was proved that mice lacking HO-1 developed progressive inflammatory diseases [47]. Upregulation of HO-1 leads to decreased expression of the nuclear factor-κB (NF-κB) pathway and the downregulation of HO-1 is linked with vulnerability to oxidative damage, augmented pro-inflammatory state, and triggered extreme cellular injury [48]. In our study, the protein expressions of HO-1 and AKT were downregulated by PD treatment. Meanwhile, L-carnitine elevated the HO-1 expression and up regulated AKT expression. Prior research demonstrated that increased expression of HO-1 could inhibit oxidative damage in parallel with prominent activity of Nrf2-ARE-binding [49, 50].

Transforming growth factor β1 (TGFβ1) is a powerful known profibrogenic cytokine which augments proliferation and differentiation of fibroblasts and deposition of extracellular matrix that cause epithelial cells injury [51]. TGF-β1 activation may be important in the early phases of acute lung injury [52]. Previous studies reported the important role of TGF-β1 in the pathophysiology of experimentally-induced pulmonary fibrosis [53]. Additionally, TGF-β increase ROS levels by inhibiting upregulation of several antioxidant enzymes including glutathione peroxidase (GPx), catalase, and superoxide dismutase (SOD) [54], and by lowering the concentration of glutathione, the most abundant intracellular free thiol [55] which cause lung injury. Our results showed that L-carnitine treatment diminished the elevated level of TGF-β1 in PD group. L-carnitine, in previous study, reduced hepatic TNF-α and TGF-β1 ameliorating of HFD-induced hepatic dysfunction [56], indicating that L-carnitine inhibits the accumulation of oxidative stress and inflammatory mediators induced by PD. Other researchers showed that the existence of TGF-β1 in the alveolar space could cause accumulation of inflammatory cells and fibroblasts, as well as stimulated deposition of connective tissue by fibroblasts [57]. L-carnitine effectively reduced the number of goblet cell, inhibited the mucus formation in bronchioles and interstitial inflammatory infiltrate as well as alleviated the destruction of alveolar walls, and the congestion of blood vessels in lung tissue induced by PD.

## 5 Conclusion

The current study highlight the antioxidant and anti-inflammatory properties of L-carnitine mediated via activating Nrf2/Keap1 signaling pathways, the modulation of HO-1/NQO1/GCLM activity and alleviating TGF-β1 expressions. Hence L-carnitine has a promising therapeutic effect for chromium-induced acute lung injury.

## Declarations

### Author contribution statement

Abeer Salama: Conceived and designed the experiments; Performed the experiments; Analyzed and interpreted the data; Contributed reagents, materials, analysis tools or data; Wrote the paper.

Hany M Fayed: Analyzed and interpreted the data; Contributed reagents, materials, analysis tools or data; Wrote the paper.

Rania Elgohary: Performed the experiments; Analyzed and interpreted the data; Contributed reagents, materials, analysis tools or data; Wrote the paper.

### Funding statement

This research did not receive any specific grant from funding agencies in the public, commercial, or not-for-profit sectors.

### Data availability statement

Data included in article/supplementary material/referenced in article.

### Declaration of interests statement

The authors declare no conflict of interest.

### Additional information

No additional information is available for this paper.
